# Evaluation of central and peripheral effects of doxepin on acetic acid-induced colitis in rat and the involved mechanisms

**DOI:** 10.17179/excli2016-727

**Published:** 2017-03-30

**Authors:** Mohsen Zabihi, Valiollah Hajhashemi, Ardeshir Talebi, Mohsen Minaiyan

**Affiliations:** 1 School of Pharmacy and Pharmaceutical Sciences, Isfahan University of Medical Sciences, Isfahan, Iran; 2 Department of Pharmacology and Isfahan Pharmaceutical Sciences Research Center, School of Pharmacy and Pharmaceutical Sciences, Isfahan University of Medical Sciences, Isfahan, Iran; 3 Department of Clinical Pathology, School of Medicine, Isfahan University of Medical Sciences, Isfahan, Iran

**Keywords:** doxepin, colitis, inflammation, antidepressants, rats

## Abstract

Anti-colitis effect of antidepressants has been demonstrated recently. Doxepin, a tricyclic antidepressant drug (TCA), with potent H_1_, H_2_, alpha_1_ adrenergic and muscarinic receptor blocking effects could be a good candidate for investigation for its anti-colitis activity. Moreover high prevalence of depression in patients who suffer from IBD (inflammatory bowel disease), defends this idea that adjuvant therapy with an antidepressant drug which has anti-inflammatory effect, may exert favorable effects in the control of the disease. In this study colitis was induced by acetic acid instillation into rat's colon. Doxepin was injected by intraperitoneal (10, 20, 40 mg/kg, twice daily, i.p.) or intracerebroventricular (50 and 100 microgram/rat, i.c.v.) routes to separate the mechanisms are absolutely exerted centrally or mediated both centrally and peripherally prior to induction of colitis. Dexamethasone (2 mg/kg/day, i.p.) was used as reference drug. All the treatments continued for three successive days. The effectiveness of drug was evaluated by determination of cytokines (TNFα, IL6 and IL1β) and myeloperoxidase (MPO) activity as well as macroscopic scores and histopathological parameters. Doxepin after i.p. administration was effective to reduce colitis severity through reduction in the macroscopic and microscopic colonic parameters, MPO activity and cytokines levels. Intracerebroventricular administration of the drug in contrast, did not show any significant protective effect suggesting no important central mechanisms for anti-colitis activity of doxepin. Doxepin as an ancient antidepressive drug has anti-colitis and anti-inflammatory properties which are mainly exerted peripherally so it could be introduced as a good candidate for depressed people who suffered from IBD disorders.

## Introduction

Inflammatory bowel disease (IBD) is a bowel disorder that distinguished by incidences of exacerbations and relieves periodically, and it includes some kinds of disorders such as ulcerative colitis (UC) and Crohn's disease (CD) (Bernstein et al., 2009[2]). Clinical manifestations of these disorders are edema and ulceration of the colon so that cause some symptoms in patient such as abdominal pain, bloody or mucosal diarrhea, fever, reduced body weight and weakness (Thoreson and Cullen, 2007[53]). Prolonged IBD can cause many psychological problems in patient (Sajadinejad et al., 2012[44]). It has shown that psychiatric disorders are more common in IBD patients compared to other people (Kurina et al., 2001[25]) and these disorders have more severe symptoms while the disease is severe (Graff et al., 2009[16]). There are high prevalence of depression (30 %) over remission period and anxiety (80 %) and depression (55 %) over relapse period (Mikocka-Walus et al., 2012[28]). In addition the risk factors for relapse are some psychiatric disorders like depression (Häuser et al., 2011[20]). The studies have shown antidepressant medications improve some symptoms of IBD such as urgency of defecation and pain (Mikocka-Walus et al., 2012[27]; Mikocka-Walus et al., 2006[29]).

Antidepressant medications have several pharmacological properties whereas their mechanisms of actions are not completely obvious (Pollack and Doyle, 2003[38]; Rahimi et al., 2009[39]; Sawynok et al., 2001[46]). Some recent investigations suggest that antidepressants produce both *in vitro* and *in vivo* anti-inflammatory activities (Hajhashemi et al., 2015[18]; Kostadinov et al., 2014[24]; Sadeghi et al., 2013[43]; Sutcigil et al., 2007[52]; Sacre et al., 2010[41]).

Our previous studies also demonstrated the anti-inflammatory activities of amitriptyline, fluvoxamine, maprotiline and venlafaxine (Hajhashemi et al., 2010[19], 2015[18]; Sadeghi et al., 2011[42], 2013[43]) and anti-colitis effects of maprotiline and fluvoxamine (Minaiyan et al., 2014[31], 2015[32]).

Doxepin, a TCA with a tertiary amine, inhibits the reuptake of norepinephrine (NE) and serotonin (5HT) and exerts a very weak inhibition of dopamine (DA) reuptake. Its active metabolite, desmethyldoxepin (nordoxepin), has also some antidepressant effects. Doxepin binds strongly to histamine H_1_ and H_2_ receptors (Shibuya et al., 2012[48]; Ahles et al., 1984[1]; Shimamura et al., 2011[49]). It also has some antagonistic effects on 5-HT, alpha_1_ adrenergic and muscarinic cholinergic receptors (Singh and Becker, 2007[50]).

Doxepin is approved for treatment of major depression (MDD) and insomnia (Wichniak et al., 2012[57]), as a part of the treatment of chronic urticaria (Negro-Alvarez et al., 1996[37]) and in pain management (Godfrey, 1996[15]; Sansone and Sansone, 2008[45]).

The roles of some nervous systems and mediators in the pathogenesis of colitis such as sympathetic nervous system (SNS) (Straub et al., 2006[51]), histamine (Fogel et al., 2005[12]; Xie and He, 2005[58]), serotonin (Ghia et al., 2009[13]; Shajib and Khan, 2015[47]) and dopamine (Tolstanova et al., 2010[54]), make doxepin as a good candidate for therapy of inflammatory conditions such as IBD.

The present study was aimed to evaluate the effect of doxepin on experimental colitis in rats. For more clear detection of peripheral and/or central roles of doxepin effect in this experiment, we applied the drug by either i.p. or i.c.v. injections.

## Materials and Methods

### Animals

Male Wistar rats weighing about 200-250 g were purchased of the animal house of the School of Pharmacy, Isfahan University of Medical Sciences, Isfahan, Iran. The rats were housed in groups of 6 in temperature and humidity controlled rooms (20-23 °C, 50-60 %) with a 12 h light/dark cycle and free access to standard food and tap water. The animals were kept and handled according to the local guidelines of care and work with laboratory animals in Isfahan University of Medical Sciences.

### Chemicals

Doxepin hydrochloride (Sigma, USA) and dexamethasone hydrochloride (Darupakhsh Company, Iran) were dissolved in isotonic saline. Formalin, glacial acetic acid and diethyl ether oxide (Merck, Darmstadt, Germany) were also used.

Dibasic potassium phosphate (Merck, Germany), monobasic potassium phosphate (Merck, Germany), hexadecyl trimethyl ammonium bromide (HTAB) and o-dianisidine dihydrochloride (o-dianisidine) (Sigma Chemical Co., St. Louis, Mo, USA), hydrogen peroxide (H_2_O_2_) was used for determination of myeloperoxidase (MPO) activity.

TNF-α, IL-6 and IL-1β kits (Boster, USA) were used for measurement of the cytokines.

Ketamine and xylazine vials (Alfasan, The Netherlands) were used for inducing anesthesia in rats. 

### Surgical procedure 

To adapt the rats to manipulation and reduction their stress, they were handled for five days. Then a mixture of ketamine (40 mg/kg) and xylazine (7 mg/kg) by i.p. injection was used to anesthetize them. Then, the rats were fixed in a stereotaxic apparatus (Stoelting, USA), and according to Paxinos and Watson rat atlas, an i.c.v. cannula was implanted (AP: −0.8 mm; LR: 1.4 mm; UD: 3.3 mm) (Budantsev et al., 1992[4]), the cannula was fixed by dental cement.

A needle (no. 22) with 2 mm length was used as cannula and a needle (no. 30) was inserted inside the cannula for the drug injection. To check the right cannula implantation, the animals were sacrificed at the end and brain tissues were tested.

### Body weight measurement

The rats were individually weighed by a digital balance (ACCU-LAB V-3000) at the start of the experiment and at the end of study in order to measure body weight changes. 

### Induction of experimental colitis

The animals were kept in stainless steel cages with a fasted and free access to water condition for about one day before colitis induction. Acute colitis was induced by acetic acid as it was described previously (MacPherson and Pfeiffer, 1978[26]). Diethyl ether as an anesthetic agent was inhaled to the rats placed in a desiccator. After the rats were lightly anesthetized, the whole of an 8 cm tube was inserted into the colon via the anus. Then acetic acid (3 ml, 3 % v/v in normal saline) was entered into the colon slowly. Then for 30 sec they were held in a head down position to decrease leakage of the solution from anus.

### Experimental design

The doses of doxepin were chosen from a pilot study. The following groups were designed and the rats were distributed into each group randomly:

Sham group (n=6): took normal saline (2 ml/kg, i.p.) without colitis induction; Control group (n=6): took normal saline (2 ml/kg, i.p.) 30 min prior to induction of colitis; Dexamethasone group (n=6): took dexamethasone (2 mg/kg, i.p.) 30 min prior to induction of colitis. Test groups (n=6): took doxepin (10, 20, 40 mg/kg twice a day, i.p. or 50, 100 μg/rat/day, i.c.v.) 30 min prior to induction of colitis.

Intracerebroventricular injections were accomplished slowly during 1 min through the cannula at the volume of 10 μl.

Administration of medications was performed for three successive days following of the colitis induction. All the drugs solutions were prepared freshly.

### Assessment of colon damage 

The animals were sacrificed in the fourth day of colitis induction by diethyl ether inhalation. The colons were separated and washed with normal saline quietly and weighed accurately (Minaiyan et al., 2015[32]). The 8 cm distal colons were assayed for macroscopic evaluations and then the colons were fragmented into three pieces to use for histopathology assessment, measuring MPO activity and cytokine levels. The tissue segments for histopathology assessment were kept in formalin 10 % and the segments for measuring MPO activity and cytokine levels were kept at freezer (-85 °C). 

### Macroscopic assessment of colon injury

The samples were photographed by digital camera (Sony, Japan) and the lesions were analyzed by a software (Fiji-win 32). Then an image processing and analysis software was done by NIH (National Institutes of Health) Image for the Macintosh (Ghosh et al., 2004[14]). For assessment the severity of colitis macroscopically, five scores were chosen by an independent viewer. Summation of the following grades were considered to calculate the macroscopic score:

Number 0 for conditions without any macroscopic alterations, number 1 for only erythema conditions on colon's mucosa tissue, number 2 for mild conditions of edema or bleeding or erosion on mucosa tissue, number 3 for moderate conditions of edema or ulcers or erosions, number 4 for severe conditions of edema or ulcers or erosions or necrosis (Deshmukh et al., 2010[5]). Furthermore, ulcer area was measured for each specimen. Ulcer index was calculated using the following equation as described by Varshosaz et al. (2010[56]).

Ulcer index = Ulcer area (cm^2^) + Macroscopic score.

### Histopathological assessment of colon injury

The colon pieces which had been fixed in formalin solution were dried and derived in paraffin and then cut into slices with 5 μm thickness. Then paraffin was cleaned by xylene solution and the slices were dyed by HE method (hematoxylin and eosin staining protocol). Finally the dyed slices were scored as described previously (Rees, 1998[40]) with some modifications. Total colitis index was measured by summing the scores of inflammation severity (0-3), inflammation extent (0-3), crypt damage (0-4) (Minaiyan et al., 2015[32]).

### Assay for MPO activity

Assessment of tissue MPO activity was carried out according to the technique described by Bradley et al. (1982[3]) with some modifications. Segments of the colon (0.1 g) were thawed in laboratory environment and crushed to prepare the suspensions. The suspensions were homogenized in potassium phosphate buffer (50 mM, pH=6) plus HTAB (0.5 %) in an ice bath. It was applied enough buffer to make suspensions with 0.1 g tissue per 5 ml. Then the suspensions were respectively sonicated (10 s, in an ice bath), worked under exposure of freezing-thawing cycle (3 times), sonicated (10 s, in an ice bath) and centrifuged (15 min, 15000 rpm at 4 °C). Finally the supernatant of each suspension was extracted. The supernatants were added to phosphate buffer (50 mM, pH 6) containing O-dianisidine dihydrochloride (0.167 mg/ml) and hydrogen peroxide (0.0005 %) to make a solution with 0.1 ml (supernatant) to 2.9 ml (buffer) ratio. The absorbance of each final solution was recorded by a spectrophotometer (EPOCH, USA) at 460 nm.

### Determination of the cytokines levels in the colon tissue

TNF-α, IL-6 and IL-1β levels in the colon tissues were evaluated by enzyme-linked immunosorbent assay (ELISA). Segments of the colon were thawed in laboratory environment and chopped to small pieces and were homogenized in 0.01 M PBS (pH=7.2-7.6) containing 8.5 g NaCl, 1.4 g Na_2_HPO_4_ and 0.2 g NaH_2_PO_4_ to 1000 ml distilled water. One ml PBS per 1 g tissue was used to prepare the supernatant. The cytokines levels were measured according to kit instructions provided by the manufacturer (Boster Company).

### Statistical analysis

Data are expressed as mean ± S.E.M which were analyzed by one-way ANOVA followed by Tukey's post hoc test. Paired t-test was applied for weigh changes comparison. All statistical analyses were made by using SPSS software (version 22). 

## Results

### Animals' body weight changes

Induction of experimental colitis caused loss of body weight during the experimental period in the control group. The rats were treated with doxepin (20, 40 mg/kg, i.p.) significantly showed improvement in body weight loss at day 4. Dexamethasone (2 mg/kg, i.p.) as reference drug resulted in improvement of body weight loss too. In Sham group, three days experiment caused body weight gain as expected (P<0.001, paired t-test) (Table 1(Tab. 1)).

### Effect of doxepin on macroscopic parameters

**Figure 1:**
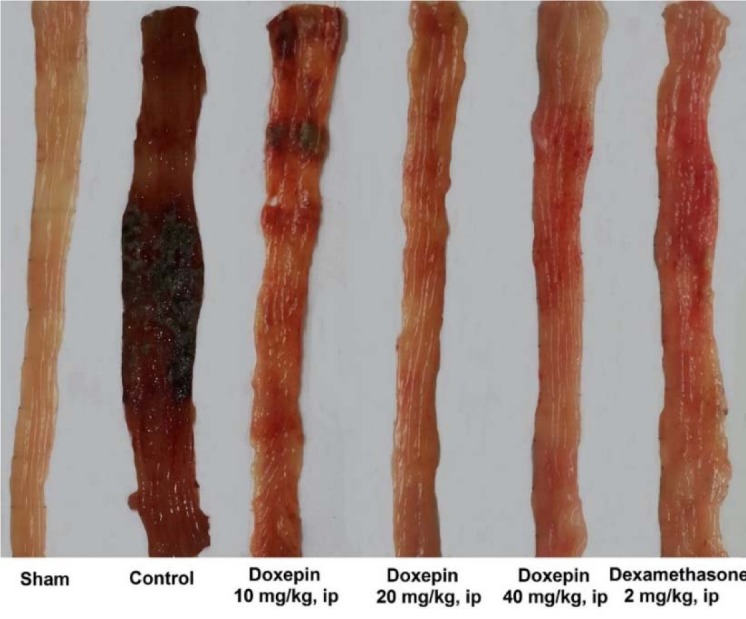
Macroscopic presentation of rat colons in treatment groups. Sham: normal rats treated with normal saline which shows intact colon, Control: control colitis treated with normal saline which shows the highest level of tissue injuries like edema, erythema, ulcer, necrosis and thickening of tissue. Doxepin and dexamethasone treated colons represents healing of ulcers and obvious improvement in tissue injuries. i.p.: intraperitoneal

**Figure 2:**
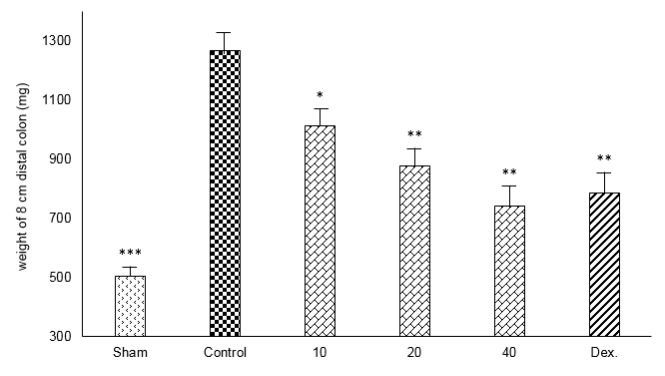
Effect of doxepin (10, 20, 40 mg/kg, i.p.) and dexamethasone (Dex., 2 mg/kg, i.p.) on weight of 8 cm distal colon. Data are analysed as mean ± S.E.M, (n=6). *P<0.05, **P<0.01, ***P<0.001 in comparison with control group, one-way ANOVA followed by Tukey's post hoc test.

**Figure 3:**
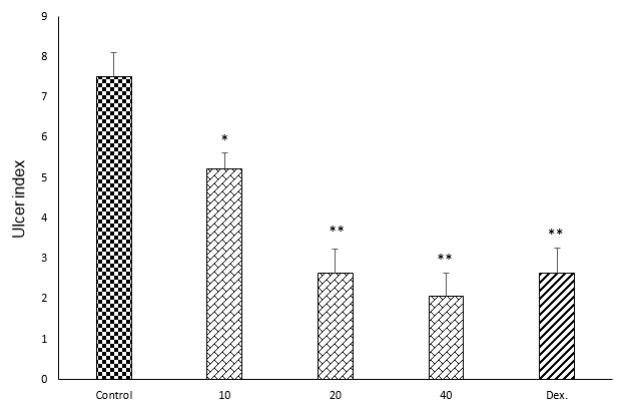
Effect of doxepin (10, 20, 40 mg/kg, i.p.) and dexamethasone (Dex., 2 mg/kg, i.p.) on ulcer index. Data are analysed as mean ± S.E.M, (n=6). *P<0.05, **P<0.01 in comparison with control group, one-way ANOVA followed by Tukey's post hoc test.

Resulting of colitis induction, the colons of control group displayed severe inflammation, ulceration, wall thickening, edema and sometimes necrosis, while colons of sham group showed intact epithelium with no damage (Figure 1(Fig. 1)). One-way ANOVA followed by Tukey's post hoc test revealed that weight of distal colon and ulcer index (summation of ulcer area and macroscopic score) were significantly increased in control group during experimental period (P<0.01). Treatment with doxepin (20 and 40 mg/kg, i.p.) reduced both weight of colon and ulcer index compared with control group (P<0.01) (Figures 2(Fig. 2), 3(Fig. 3)). With the dose of 10 mg/kg, doxepin reduced ulcer index and colon weight in less amount compared to its other doses (p<0.05). The effects of doxepin (20 and 40 mg/kg, i.p.) was nearly similar to dexamethasone (2 mg/kg, i.p.) (Figures 2(Fig. 2), 3(Fig. 3)).

**Figure 4:**
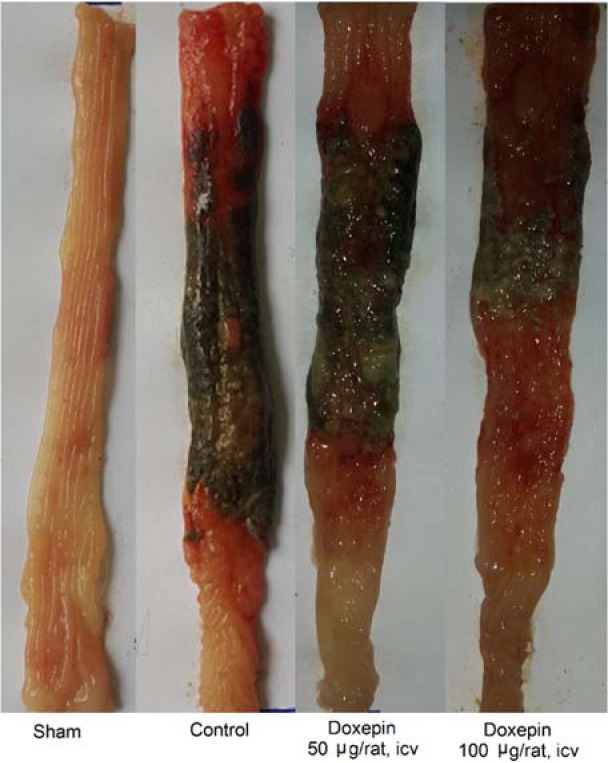
Macroscopic presentation of rat colons in treatment groups. Sham: normal rats treated with normal saline which shows intact colon, Control: control colitis treated with normal saline which shows the high level of tissue injuries like edema, erythema, ulcer, necrosis and thickening of tissue. Doxepin: treated colitis with doxepin (50 and 100 µg/rat. i.c.v.) which both of them show high level of tissue injuries like control colitis. i.c.v.: intracerebroventricular injection.

**Figure 5:**
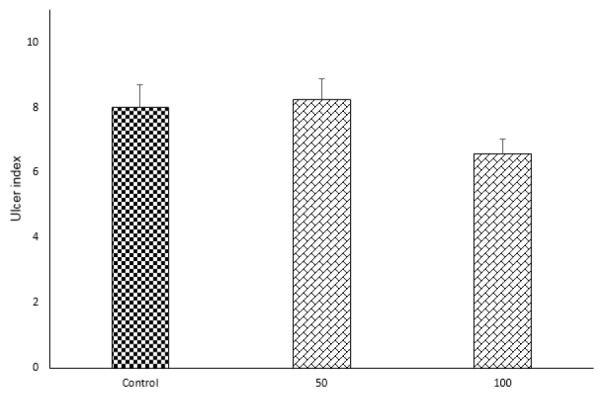
Effects of doxepin (50, 100 μg/rat, i.c.v.) on ulcer index. Results are presented as mean ± S.E.M, (n=6).

Doxepin (50 μg/rat and 100 μg/rat) after i.c.v. injection did not show any significant anti-colitis activity in rats in comparison with the control group (p<0.05) (Figures 4(Fig. 4) and 5(Fig. 5)). 

### Effect of doxepin on histopathological features

**Figure 6:**
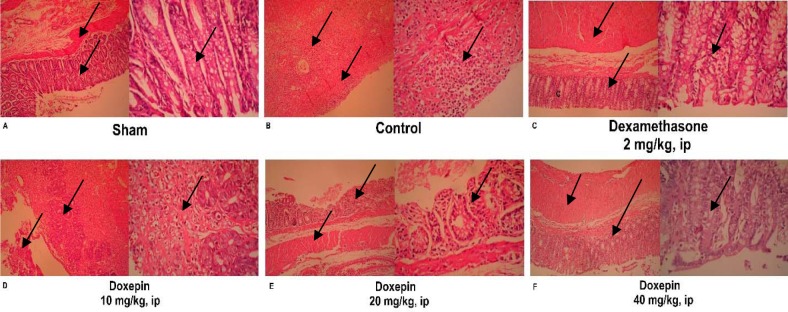
Histopathological presentation of rat colons in treatment groups: (A) Appearance of a normal rat colon. (B) In control colitis group, great mucosal layer destruction with most inflammatory cell infiltration and cryptic damage are evident. (C) In dexamethasone group, the extent and severity of histological damage were attenuated while the goblet cells could be seen. (D, E, F) Improvements in histopathology damage parameters were obvious especially with greater dose of doxepin (40 mg/kg).

There was not any histological damage in sham group, so colonic mucosa had an intact epithelium. In contrast, control group showed edema, inflammation (existing of various inflammatory cells), trans-mural ulceration, cell infiltration into the mucosa and exfoliated and destroyed epithelium. Interestingly, goblet cells have been seen only in dexamethasone group unlike to doxepin groups (Figure 6(Fig. 6)).

**Figure 7:**
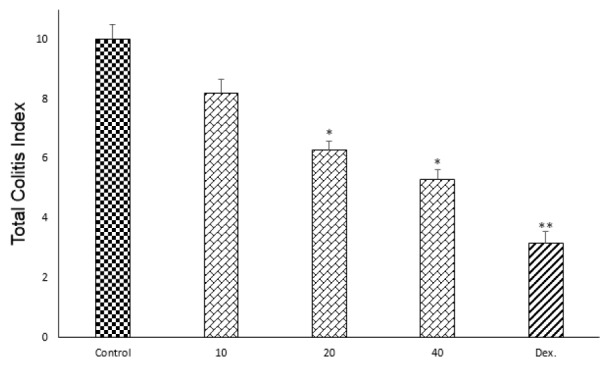
Effect of doxepin (10, 20, 40 mg/kg, i.p.) and dexamethasone (Dex., 2 mg/kg, i.p.) on total colitis index. Data are analysed as mean ± S.E.M, (n=6). *P<0.05, **P<0.01 in comparison with control group, one-way ANOVA followed by Tukey's post hoc test.

One-way ANOVA followed by Tukey's post hoc test explained that total colitis index, including summation of inflammation severity, inflammation extent and crypt damage scores were significantly decreased in doxepin (20 and 40 mg/kg, i.p.) and dexamethasone (2 mg/kg, i.p.) groups in comparison with control groups (p<0.05), without any significant improvement with doxepin at 10 mg/ kg, i.p. dose (Figure 7(Fig. 7)). 

### Effect of doxepin on myeloperoxidase (MPO) activity

**Figure 8:**
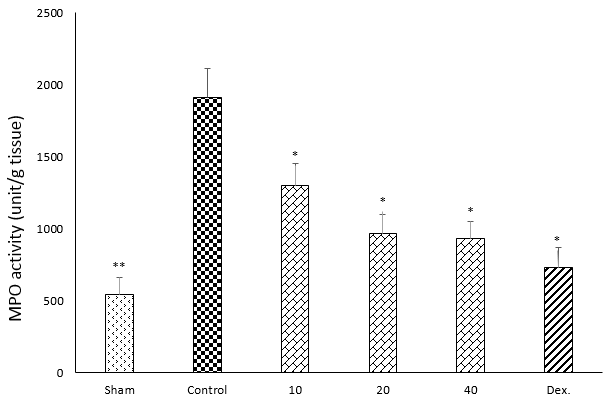
Effect of doxepin (10, 20, 40 mg/kg, i.p.) and dexamethasone (Dex., 2 mg/kg, i.p.) on MPO activity of rat colon. Data are analysed as mean ± S.E.M, (n=6). *P<0.05, **P<0.01 in comparison with control group, one-way ANOVA followed by Tukey's post hoc test.

One-way ANOVA followed by Tukey's post hoc test revealed that MPO activity in colonic tissue of the control group was significantly increased (P<0.01) in comparison with sham group. Myeloperoxidase activity in colonic tissues of dexamethasone group and all of doxepin groups (P<0.01) decreased in comparison with control group. Indeed, three increasing doses of doxepin had nearly the same effect on the level of MPO (Figure 8(Fig. 8)).

### Effect of doxepin on the tissue cytokines

**Figure 9:**
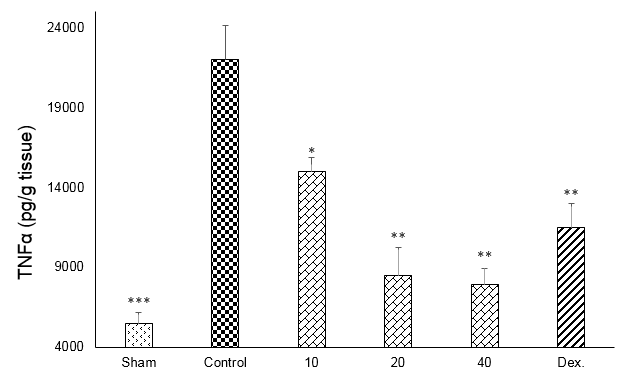
Effect of doxepin (10, 20, 40 mg/kg, i.p.) and dexamethasone (Dex., 2 mg/kg, i.p.) on TNF-α. Data are analysed as mean ± S.E.M, (n=6). *P<0.05, **P<0.01, ***P<0.001 in comparison with control group, one-way ANOVA followed by Tukey's post hoc test.

**Figure 10:**
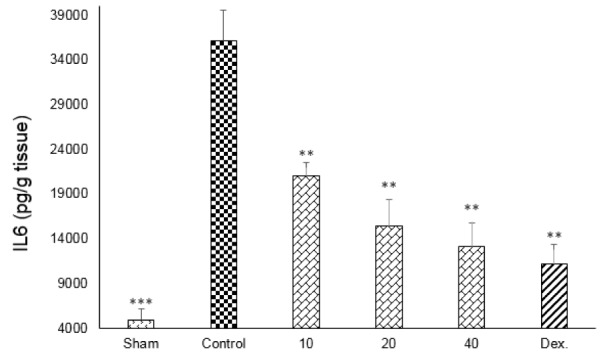
Effect of doxepin (10, 20, 40 mg/kg, i.p.) and dexamethasone (Dex., 2 mg/kg, i.p.) on IL6. Data are analysed as mean ± S.E.M, (n=6). **P<0.01, ***P<0.001 in comparison with control group, one-way ANOVA followed by Tukey's post hoc test.

**Figure 11:**
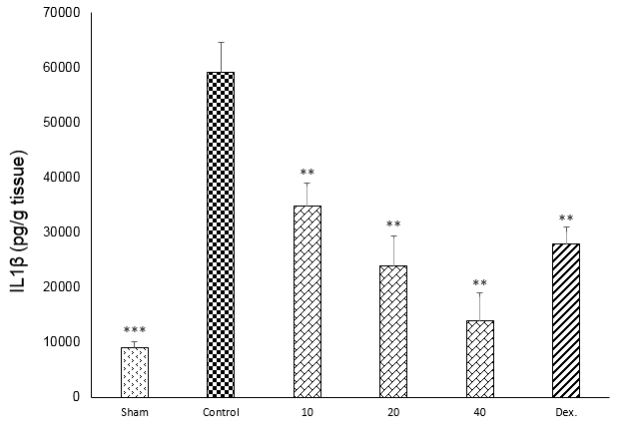
Effect of doxepin (10, 20, 40 mg/kg, i.p.) and dexamethasone (Dex., 2 mg/kg, i.p.) on IL1β. Data are presented as mean ± S.E.M, (n=6). **P<0.01, ***P<0.001 in comparison with control group, one-way ANOVA followed by Tukey's post hoc test.

One-way ANOVA with Tukey's post hoc test showed that intracolonic instillation of acetic acid significantly (p<0.01) increased tissue levels of TNF-α, IL-6 and IL-1β in comparison with sham group (Figures 9-11(Fig. 9)(Fig. 10)(Fig. 11)).

All three tested doses of doxepin (10, 20 and 40 mg/kg twice daily, i.p.) and dexamethasone (2 mg/kg, i.p.) could decrease these cytokines (p<0.01) (Figures 9-11(Fig. 9)(Fig. 10)(Fig. 11)). However, there was no significant difference between 20 and 40 mg/kg doses of doxepin for TNFα attenuation, two greater doses of 20 and 40 mg/kg were more effective than the dose of 10 mg/kg in this respect (Figure 9(Fig. 9)).

## Discussion

In previous studies it has figured out that antidepressants can inhibit inflammatory disorders (Hajhashemi et al., 2010[19], 2015[18]). *In vitro* studies have shown that some antidepressants reduce stimulated release of pro-inflammatory cytokines such as IL-1β, IL6, TNF-α and IFN-γ (Janssen et al., 2010[22]). Sutcigil et al. (2007[52]) found a decrease in elevated TNF-α levels after selective serotonin reuptake inhibitors (SSRI). Sadeghi et al. (2011[42]) showed that amitriptyline, as a TCA, reduced levels of IL-1β, TNF-α and MPO activity into inflamed paw tissues. Interestingly, Hinze-Selch et al. (2000[21]) reported that therapy with TCAs, but not SSRIs, activated the TNF-α system in patients. Hence, study on the effects of antidepressant drugs on the cytokine levels and MPO activity is one of the experimental issues now. MPO activity acts as an indicator for oxidative stress magnitude in involved tissues. Decline in MPO activity after doxepin treatment suggests that antioxidant capacities of colonic tissues are restored after treatment by different doses of doxepin (Sadeghi et al., 2011[42]).

There are some studies to explain the beneficial outcome of antidepressant medications in IBD suffers. In a study, fluoxetine and desipramine showed anti-inflammatory effect in acetic-acid induced colitis in rats (Guemei et al., 2008[17]). Anti-inflammatory effects of fluvoxamine (Minaiyan et al., 2015[32]), amitriptyline (Fattahian et al., 2016[9]) and maprotiline have investigated by Minaiyan et al. (2014[31]) in acetic-acid induced colitis in rats.

Doxepin as a TCA with some properties like H_1_, H_2_ and muscarinic receptor antagonistic activity was chosen as a suitable antidepressant for evaluation.

To make a distinguish difference between its peripheral and central effects; the study was done by i.p. and i.c.v. administration of doxepin respectively. For this purpose, we accomplished a pilot study to determine an appropriate dose range. Then the doses of 10, 20 and 40 mg/kg twice daily for i.p. administration and 50 and 100 μg/rat for i.c.v. administration which were both effective and safe were chosen.

The findings clearly explained that doxepin can inhibit colitis parameters in the various applied i.p. doses and it has a potent anti-inflammatory effect in acetic acid induced colitis in rat. It improved all of the colitic markers at applied doses.

Intra-cerebraventricular (i.c.v.) administration of doxepin didn't show any significant effect on colitis. It means that peripheral mechanisms are necessary for doxepin action or activation in colitis improvement. It is notable that doxepin's active metabolite, desmethyldoxepin (nordoxepin), with antidepressant effect might be responsible at least for a part of doxepin effectiveness on colitis. Also doxepin by itself has some specific properties that can explain its peripheral effectiveness on colitis. It binds strongly to the histamine H_1_ and H_2_ receptors (Shibuya et al., 2012[48]; Ahles et al., 1984[1]; Shimamura et al., 2011[49]) and has some antagonistic effects on 5-HT, alpha_1_ adrenergic and muscarinic receptors (Singh and Becker, 2007[50]). The roles of these mediators in the pathogenesis of colitis have been previously investigated (Fogel et al., 2005[12]; Ghia et al., 2009[13]; Tolstanova et al., 2010[54]; Xie and He, 2005[58]).

We know that immune system dysregulation has an important role in colitis pathogenesis, so it may be possible that doxepin can alter the immunity system by interacting with SNS main neurotransmitter, NE (Elenkov et al., 2000[6]; Nance and Sanders, 2007[36]).

Some studies have indicated that transporters of 5HT and NE are expressed on mononuclear cells in peripheral blood circulation besides the CNS (Faraj et al., 1994[8]; Fazzino et al., 2008[10]; Urbina et al., 1999[55]). Furthermore, immune cells such as lymphocytes and monocytes release serotonin and noradrenaline (Finocchiaro et al., 1998[11]; Mossner and Lesch, 1998[33]). Thus, it is possible that the inhibitory effect of doxepin on the levels of cytokines in colitis tissues is resulted from its direct effects on the immune cells.

Koh et al. (2011[23]) showed that fluoxetine can directly inhibit NF-κβ signaling in intestinal epithelial cells (IEC) and ameliorate experimental colitis. So inhibition of NF-κB signaling could be one of the probable mechanisms which doxepin decreases inflammatory mediators from the intestinal immune cells. Therefore assessment of NF-κB could be recommended in further experiments.

Recent studies have declared the role of serotonin and its receptors in activation of immune responses and inflammation (Shajib and Khan, 2015[47]). Serotonin receptor (5HT3) inhibition by ondansetron (Motavallian-Naeini et al., 2012[35]), granisetron (Fakhfouri et al., 2010[7]), tropisetron (Motavallian et al., 2013[34]) or ramosetron (Min and Rhee, 2015[30]) has beneficial property on experimental colitis in rat. It has figured out that there are serotoninergic receptors especially 5HT3 ones in immune system, so they have a significant role in infiltration and activation of macrophages into the inflamed intestine. Regarding to antagonistic effects of doxepin on 5HT1 receptors, its anti-colitis effects could be attributed somewhat to its antihistaminic activity.

Our findings are supportive for the studies investigating the role of antidepressants in management of depressed mood as well as visceral inflammation in IBD patients. Periodic monitoring of IBD suffers for depressive disorders, is supportive to adjuvant therapy with an antidepressant. Also our findings reveal that doxepin could be a favorite candidate for relieve the comorbidities related to depression in patients with IBD, such as insomnia. Further studies are needed to introduce doxepin as a safe and effective drug in prevention and/or treatment of IBD.

## Acknowledgements

This study was financially supported by Vice Chancellor of Research, Isfahan University of Medical Sciences, Isfahan, I.R. Iran.

## Figures and Tables

**Table 1:**
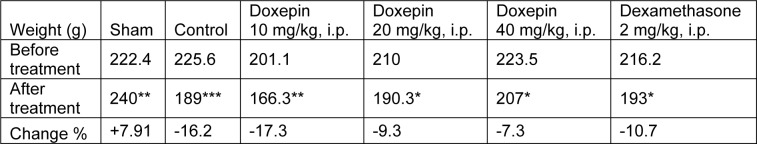
Effect of doxepin on body weight reduction before and after treatment. Data are presented as mean ± S.E.M (n=6). *P<0.05, **P<0.01, ***P<0.001, paired t-test.
